# Empowering Patients Through Literacy: A Randomized Controlled Trial of an Educational App for Evaluating Complementary Therapies in Diabetes Management

**DOI:** 10.1002/nop2.70658

**Published:** 2026-06-26

**Authors:** Hsiao‐Yun Chang, Kuei‐Chun Yeh, Yu‐Yao Huang, Jui‐Hsiang Li, Feng‐Hsuan Liu

**Affiliations:** ^1^ Department of Nursing Chang Gung University of Science and Technology Taoyuan Taiwan; ^2^ Division of Endocrinology and Metabolism, Department of Internal Medicine Linkou Chang Gung Memorial Hospital Taoyuan Taiwan; ^3^ Division of Endocrinology and Metabolism, Department of Internal Medicine Taoyuan General Hospital Taoyuan Taiwan; ^4^ College of Medicine Chang Gung University Taoyuan Taiwan

**Keywords:** communication, complementary therapy, decision‐making, diabetes, health literacy, quality of life, self‐efficacy

## Abstract

**Aims:**

This study evaluated the effectiveness of an Educational App for Complementary Therapy in Diabetes Management in improving health literacy, quality of life, and diabetes empowerment through complementary therapy.

**Design:**

An assessor‐ and investigator‐blinded randomized controlled trial was conducted. The report of this study adheres to the CONSORT 2025 guideline.

**Methods:**

Eighty‐eight participants with type 2 diabetes who reported complementary therapy use were randomly assigned to either an app‐based intervention or a standard‐education control group. The eight‐session intervention incorporated self‐control, cognitive, psychological, and behavioural strategies. Outcomes, including diabetes empowerment, complementary therapy health literacy, and quality of life, were assessed at three time points. Data were analysed using generalized estimating equations.

**Results:**

Participants in the intervention group demonstrated statistically significant improvements in CT‐related critical health literacy and quality of life compared with the control group, but no statistically significant improvement in diabetes empowerment. Results suggest that the educational app enhances individuals' knowledge and capacity to evaluate the use of complementary therapies.

**Conclusions:**

The Educational App for Complementary Therapy in Diabetes Management is a promising digital tool for improving complementary therapy health literacy and quality of life in diabetes care. Although empowerment did not improve, the app's design and alignment with empowerment processes highlight its potential for extended educational and clinical use. Future research should explore longer‐term engagement, methods of reinforcement, and broader implementation across diverse populations.

**Implications for the Profession and/or Patient Care:**

Healthcare professionals can utilize the app to enhance patient education on the use of complementary therapies, thereby improving communication, shared decision‐making, and the overall quality of diabetes management.

**Impact:**

What problem did the study address? The use of complementary therapy alongside conventional diabetes care is common, yet individuals often lack adequate knowledge to evaluate its potential benefits, risks, and safety. Disclosure of complementary therapy use to healthcare professionals is frequently low, posing risks of adverse interactions and undermining coordinated care. Knowledge, health literacy, and a supportive environment are critical components of chronic disease management, but they are underexplored in the context of digital interventions focused on the use of complementary therapies. What were the main findings? This study evaluates the Educational App for Complementary Therapy in Diabetes Management for smartphones—a digital educational intervention designed to enhance complementary therapy health literacy and safe decision‐making among individuals diagnosed with diabetes. The intervention demonstrated statistically significant improvements in complementary therapy health literacy and quality of life, underscoring its potential clinical utility. While diabetes empowerment did not improve within the study period, the intervention's grounding in the WHO empowerment process framework and interactive features suggest that longer or more reinforced exposure may yield stronger health outcomes. Where and on whom will the research have an impact? The app empowers individuals to make safer and more informed decisions regarding complementary therapy use, improving self‐management and quality of life. The findings provide a practical digital tool to support safer CT‐related decision‐making by helping participants appraise CT information, evaluate benefit–risk, and communicate with healthcare professionals. The research supports the integration of digital health literacy interventions into diabetes management programs, promoting evidence‐based and patient‐centred practice.

**Reporting Method:**

This study was reported in accordance with the CONSORT 2025 statement.

**Patient and Public Involvement:**

Professional experts contributed to the co‐design of the intervention. The intervention was pilot‐tested with patients, whose feedback informed protocol refinements to enhance feasibility and acceptability.

**Trial Registration:**

ClinicalTrials.gov identifier: NCT06317584

## 1 Introduction & Background

Diabetes mellitus continues to pose a significant challenge to global health, with a current estimated prevalence of 11.1% among adults aged 20–70 years. Projections indicate this figure will rise to 13.0% within the next quarter‐century (International Diabetes Federation (IDF) 2025). Notably, low and middle‐income regions bear a disproportionate burden of diabetes. For instance, Taiwan, a middle‐income country in the Western Pacific region, currently reports an adult diabetes prevalence of 13.7%, surpassing the projected global average (IDF 2025). This upward trend underscores the urgent need for comprehensive diabetes management within healthcare systems.

Conventional diabetes management primarily centres on pharmacotherapy, serum glucose monitoring, and lifestyle modifications. Recently, there has been increasing interest in the integration of complementary therapies as adjuncts to standard care, a practice that remains prevalent among individuals with diabetes (Alzahrani et al. 2021). Complementary therapy (CT) encompasses non‐mainstream health practices used in conjunction with conventional medicine, including nutritional, psychological, and physical interventions, as well as traditional systems of medicine such as Traditional Chinese Medicine and Ayurveda (National Center for Complementary and Integrative Health 2021).

In Taiwan, despite the high prevalence of CT use among individuals with diabetes—particularly the use of nutritional supplements—many patients do not disclose their use to healthcare providers (Chang et al. 2023). Moreover, patients often rely on non‐professional or word‐of‐mouth sources when engaging in CT use, reflecting a limited understanding of associated risks and benefits (Chang 2025). These practices elevate the risk of inappropriate CT use, herb–drug interactions, and compromised patient safety in diabetes management. Collectively, these concerns underscore the urgent need to enhance patient education initiatives, improve CT‐related critical health literacy, and cultivate effective patient‐provider communication to optimize diabetes care and advance comprehensive care strategies.

CT health literacy refers to the ability to obtain, process, and comprehend information relevant to complementary therapy, representing a critical skill in chronic disease self‐management (Dehghan et al. 2023). Rather than advocating for or against CT use, informed engagement requires patients to evaluate information quality, communicate effectively with healthcare professionals, and make decisions that reflect their health needs and personal values. This focus aligns closely with the concept of critical health literacy, which emphasizes the appraisal of information credibility and the application of health information in decision‐making (Tian et al. 2025). Among individuals with diabetes, CT‐related critical health literacy is particularly important because patients often encounter information from commercial, informal, traditional, and biomedical sources while also needing to consider safety risks, including herb–drug interactions. Insufficient health literacy may therefore hinder patients' ability to assess risks, seek informed guidance, and make prudent decisions about CT use, underscoring the need for targeted educational interventions to support informed appraisal and safer decision‐making about CT use alongside conventional diabetes care (Yukawa et al. 2017).

Educational strategies designed to empower individuals with diabetes to exert greater control over their health decisions are fundamental to effective diabetes care, as they enhance self‐efficacy, autonomy, and engagement in shared decision‐making (Duarte‐Diaz et al. 2023). Such strategies equip individuals with the knowledge and skills necessary for the improvement of patients' ability to access, appraise, and apply CT‐related information into diabetes management regimens. Furthermore, digital health interventions have shown promise in enhancing self‐management and empowerment among individuals with chronic diseases, leading to improved knowledge, communication with healthcare professionals, self‐efficacy, quality of life, and overall engagement (Naef et al. 2023). Although evidence suggests that digital interventions can strengthen health literacy (Verweel et al. 2023), existing CAM‐related eHealth technologies have largely emphasized safety surveillance, adverse event reporting, and the identification of potential CAM–drug or CAM–CAM interactions. In a scoping review, Ng et al. (2020) identified 69 such technologies, highlighting the growing role of digital tools in CAM safety. However, few patient‐facing, theory‐based apps have been designed to help individuals with diabetes critically appraise CT‐related information, evaluate potential benefits and risks, and communicate with healthcare professionals about CT use alongside conventional care.

Research on diabetes self‐management apps suggests that digital tools do not automatically translate into empowerment. In an analysis of 121 diabetes apps, Brew‐Sam and Chib (2019) found that most apps offered only a narrow range of features corresponding to empowerment indicators, particularly with limited customization and limited support for healthcare professional communication or social support. Their work highlights the need to explicitly connect app features with empowerment‐related mechanisms, including perceived relevance, competence, self‐determination, shared decision‐making, and patient–provider communication. In the context of CT use among individuals with diabetes, such linkage is especially important because patients need not only general diabetes self‐management support but also the ability to appraise CT‐related information, evaluate benefit–risk, and communicate safely with healthcare professionals.

To address this existing gap, the research team developed and validated the Educational App for Complementary Therapy in Diabetes Management (ECT‐Diabetes App) (Chang and Huang 2025), designed to strengthen CT‐related critical health literacy among individuals with diabetes. Rather than emphasizing specific CT modalities, the app guides users in the critical evaluation of the safety, credibility, and appropriateness of non‐mainstream practices in conjunction with conventional diabetes care. The app's design is informed by four components identified by the World Health Organization (WHO 2009) as fundamental to patient empowerment, which have been widely referenced in subsequent studies (Stepanian et al. 2023) and further adapted in Chang's (2019) empowerment model. These four strategies—self‐control, cognitive, psychological, and behavioural—serve to foster health consciousness, facilitate the acquisition of critical knowledge, and enable active patient participation in healthcare decisions impacting health outcomes.

## 2 The Study

The primary aim of this study was to evaluate the effectiveness of the ECT‐Diabetes App strengthening CT‐related critical health literacy, particularly participants' ability to understand, appraise, and apply benefit–risk information about CT use, and to examine its effects on diabetes empowerment and quality of life. We hypothesized that, compared with the control group, participants in the intervention group using the ECT‐Diabetes App would: (1) demonstrate greater improvements in CT‐related critical health literacy, (2) report higher diabetes empowerment scores, and (3) report higher perceived quality of life scores at follow‐up.

## 3 Methods

### 3.1 Study Design

This study employed an assessor‐ and investigator‐blinded randomized controlled trial with a pretest‐posttest design, conducted at hospital clinics in northern Taiwan from July 2024 to January 2025. Because participants received either an app‐based intervention or printed/verbal educational materials, true participant blinding to the delivery format was not feasible. To minimize bias, the chief investigator, outcome assessors, and statistician were blinded to group allocation. This trial was registered at ClinicalTrials.gov/NCT06317584. The study adhered to the CONSORT (Consolidated Standards of Reporting Trials) guidelines, which ensure methodological rigour and transparency in reporting findings (Schulz et al. 2011).

### 3.2 Participants and Sample Size

Recruitment took place at the outpatient diabetes clinics of two hospitals, where trained research staff distributed recruitment leaflets. Interested individuals underwent in‐person eligibility screening and a detailed assessment to confirm adherence to inclusion criteria, which included: (1) willingness to provide written informed consent; (2) diagnosis of Type 2 diabetes mellitus for a minimum of 12 months; (3) usage of complementary and alternative therapies for at least 3 months; and (4) individuals aged 20 years or older. Exclusion criteria comprised (1) non‐ownership of a smartphone; (2) visual or auditory impairments that would hinder engagement with the intervention; (3) severe medical conditions that would interfere with study participation; or (4) inability to read or write Mandarin Chinese. Participants retained the right to withdraw from the study if they experienced a serious medical event during the intervention or follow‐up period, or if they opted to discontinue participation at any point.

Given the lack of prior studies encompassing the collective concepts of individuals' knowledge acquisition about CT, interaction with healthcare professionals, CT‐related critical health literacy, and social support, the effect size was derived from a meta‐analysis that indicated digital interventions were more effective than usual care for health literacy, with a standardized mean difference of 1.22, a 95% confidence interval (CI) of 0.55–1.89 and *p* < 0.001, suggesting a large effect size (Verweel et al. 2023). The estimated sample size was 64 participants, with 32 allocated per group, calculated using G*Power for ANCOVA with main effects and interactions. This analysis yielded an effect size (*f*) of 0.4, an alpha level of 0.05, a power (1 − *β*) of 0.80, two groups, three covariates, and an allocation ratio of 1 (N_2_/N₁). After considering an estimated attrition rate of 20% (Dumville et al. 2006), the final recruitment target was established at 80 participants.

### 3.3 Randomization, Allocation Concealment, and Blinding

Following the screening assessment, eligible participants were randomly assigned to either the app group (ECT‐Diabetes App) or the control group (standard diabetes education group) at a 1:1 ratio, using a computer‐generated random allocation schedule to ensure an equal number of participants in each group. An independent statistician, blinded to the recruitment, evaluation, and intervention process, managed the random allocation sequence.

To uphold allocation concealment, the random assignments were sealed in opaque envelopes and opened only after formal enrollment. Participants were informed that they would receive education on diabetes management and CT use; however, because the intervention formats differed, participants could not be fully blinded after allocation. Only the intervention coordinators had access to group assignments. The chief investigator, outcome assessors, and statistician remained blinded to allocation, and outcome assessments were conducted entirely online to further minimize potential bias. To mitigate the risk of contamination arising from social interaction between participants, educational sessions were conducted one‐on‐one in private settings. Each site was randomly assigned to either an intervention or a control group, thereby ensuring a controlled and unbiased study environment.

### 3.4 Intervention

An initial face‐to‐face session was conducted with both intervention and control groups by a trained research nurse. This introductory session emphasized CT‐related critical health literacy, including safety considerations and the importance of communicating with healthcare providers about CT use. For the intervention group, the ECT‐Diabetes App (Version 1.0), developed by Chang and Huang (2025), was installed on participants' smartphones, while control group participants received comparable information through printed and verbal educational materials.

**FIGURE 1:**
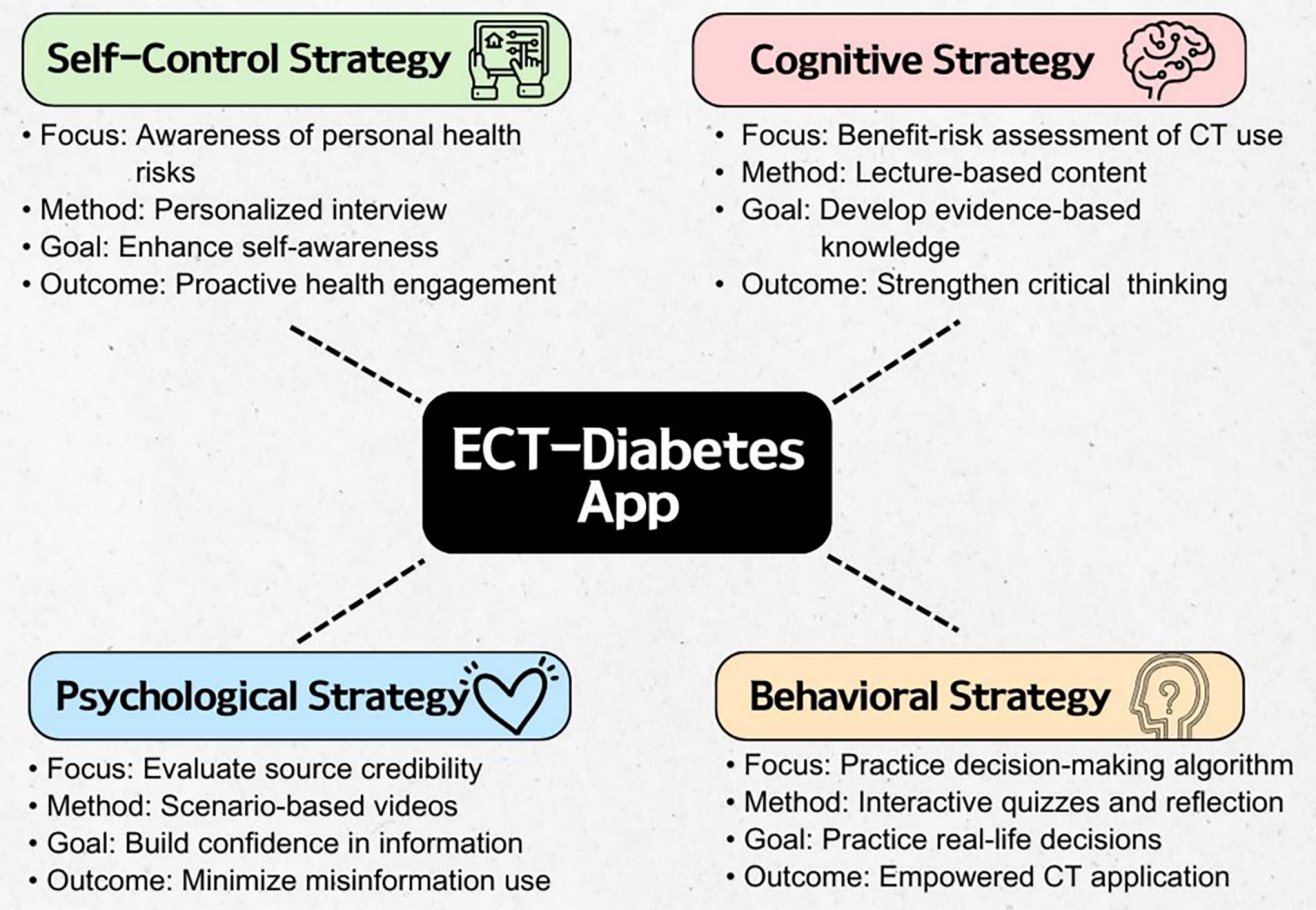
The intervention of the educational app for complementary therapy in diabetes management.

The ECT‐Diabetes App was designed as a theory‐based educational intervention to strengthen CT‐related critical health literacy among individuals with diabetes. Its purpose was not to endorse, teach, or promote specific CT modalities, but to cultivate users' ability to evaluate the safety, credibility, and appropriateness of CT‐related information in the context of conventional diabetes care. Content development was informed by a two‐round Delphi study involving 23 experts in CT, diabetes education, and health literacy. Usability testing with end‐users was subsequently conducted, and all materials underwent expert peer review to ensure medical accuracy, clarity, and patient relevance (Chang and Huang 2025). Drawing on the WHO's empowerment principles, the app operationalized four domains–self‐control, cognitive, psychological, and behavioural strategies (Chang 2020). In line with prior work (Brew‐Sam and Chib 2019) emphasizing the need to examine how specific app features correspond to empowerment‐related processes, the intervention was designed to link each feature to a hypothesized mechanism of change. Each empowerment strategy was operationalized as follows (Figure 1):

- Self‐control strategy: Aligned with the domain of health consciousness, this strategy emphasized the importance of personal health awareness. It was operationalized through a personalized face‐to‐face interview during the app orientation, prompting participants to reflect on their individual health profiles in relation to their use of CT. This approach aimed to increase self‐awareness, promote wellness‐oriented thinking, and foster proactive engagement in decision‐making related to CT.
- Cognitive strategy: Corresponding to the development of health knowledge, this strategy focused on improving participants' ability to assess the benefits and risks associated with CT use. Implementation involved evidence‐informed, lecture‐based educational content embedded within the app, designed to build foundational knowledge, improve safety awareness, and strengthen critical thinking for evaluating CT claims and using them safely alongside diabetes care.
- Psychological strategy: Centred on health credibility, this strategy sought to develop participants' confidence in evaluating information quality. It was delivered via scenario‐based video activities that trained users to identify trustworthy sources, interpret product labeling, and handle misinformation, thereby reducing reliance on unverified information and supporting safer, evidence‐based decision‐making.
- Behavioural strategy: Addressing behavioural intention, this strategy provided participants with opportunities to apply decision‐making algorithms in simulated real‐life situations. Through interactive quizzes and reflective exercises, users practiced benefit–risk assessments related to the use of CT, reinforcing structured decision‐making and empowering them to apply their knowledge in practical, health‐related contexts.

The intervention consisted of eight sequential app‐based educational sessions completed over one month. The sessions progressed from awareness to application, covering CT‐related critical health literacy, reflection on personal CT use and health status, evidence searching, credibility evaluation, product and label assessment, safety risk identification, benefit–risk assessment, and application to simulated CT‐use scenarios. Rather than promoting specific CT practices, the app emphasized information appraisal, source evaluation, safety awareness, and structured decision‐making to support safer decision‐making about CT use alongside diabetes management. Content was delivered asynchronously through short text explanations, instructional videos, scenario‐based examples, web‐based search tasks, quizzes, reflective exercises, and interactive benefit–risk assessment algorithms. These algorithms guided users to consider their medical condition, current diabetes treatment, CT product information, potential safety concerns, including herb–drug interactions, and the need to consult healthcare professionals before using CT. The app did not include automated push notifications; instead, engagement was supported through brief LINE messages during the first month and monthly 15–20 min phone calls during the second and third months to reinforce learning, address questions, and support continued application of the benefit–risk assessment process.

The app focused on CT‐related information appraisal and decision‐making rather than physiological monitoring; therefore, it did not include blood glucose monitoring tools, medication adherence logs, diet or exercise tracking, or other diabetes self‐management logs. Educational materials were accessible offline after the initial download, although web‐based search tasks required internet access. The app did not automatically collect usage analytics, such as login frequency, time spent in each session, module completion, quiz performance, or frequency of use of the benefit–risk assessment algorithms. Therefore, participant engagement was monitored through self‐report and brief follow‐up contacts rather than through built‐in app‐tracking functions. During weekly LINE contacts in the first month and monthly phone calls in the second and third months, research nurses asked participants whether they had accessed the assigned session materials, completed the reflective exercises and quizzes, and used the benefit–risk assessment steps when considering CT‐related information. Completion of the eight educational sessions within the first month was treated as the operational adherence indicator for intervention exposure. These adherence data were used for monitoring and support during the trial but were not sufficiently objective or granular to support dose–response analyses.

### 3.5 Outcomes and Measuring Instruments

#### 3.5.1 Sample Characteristics

Assessing data related to demographic characteristics, including age, gender, education, marital status, and employment status, coupled with clinical information, such as the duration of diabetes, frequency of clinical visits, current diabetes treatment, and relevant laboratory data (e.g., serum glucose levels), was essential for establishing baseline comparability and evaluating the homogeneity between the two study groups.

#### 3.5.2 Primary Outcomes

##### 3.5.2.1 CT‐Related Critical Health Literacy

The 15‐item Understanding the Benefits‐Risks of Complementary Therapies Use Scale, developed by Chang et al. (2026), measures individuals' understanding of benefit–risk management about CT use. This scale consists of four dimensions: the individual's medical condition for CTs' use, the benefit–risk assessment of CT use, the suitability of CT use, and support from healthcare professionals. Participants rate each item on a 5‐point Likert scale, ranging from 1 (strongly disagree) to 5 (strongly agree). Higher total scores indicate a more comprehensive understanding of the benefit–risks associated with CT use in diabetes management. The scale has demonstrated construct validity (Chang et al. 2026) and strong internal consistency (Cronbach's *α* > 0.90) across three time points in this study.

##### 3.5.2.2 Diabetes Empowerment

The 10‐item Diabetes Empowerment Scale, developed by Shiu et al. (2012), is designed to assess individuals' perceived control over diabetes management and their readiness for behavioural change. The scale comprises five dimensions: overcoming barriers, identifying effective management strategies, achieving goals, seeking support, and coping. Participants rate each item on a 5‐point Likert scale where 1 represents ‘strongly disagree’ and 5 indicates ‘strongly agree’. Higher total scores reflect greater self‐efficacy in diabetes self‐management. The scale has demonstrated acceptable reliability and construct validity, exhibiting strong internal consistency (Cronbach's *α* > 0.90) across three time points in the current study.

##### 3.5.2.3 Quality of Life

The 15‐item Taiwan version of the Diabetes Quality of Life Questionnaire, developed by Liu et al. (2010), is designed to assess individuals' satisfaction with various aspects of life while managing diabetes. Each item is rated on a 5‐point scale, ranging from 1 (strongly dissatisfied) to 5 (strongly satisfied), resulting in total scores that range from 15 to 75, with higher scores indicating a better‐perceived quality of life. The instrument has demonstrated validity and reliability across diverse diabetic populations. In the present study, the internal consistency of the scale was high, with Cronbach's alpha values exceeding 0.8 at three different time points, indicating robust reliability over time.

### 3.6 Data Collection and Ethics

**FIGURE 2:**
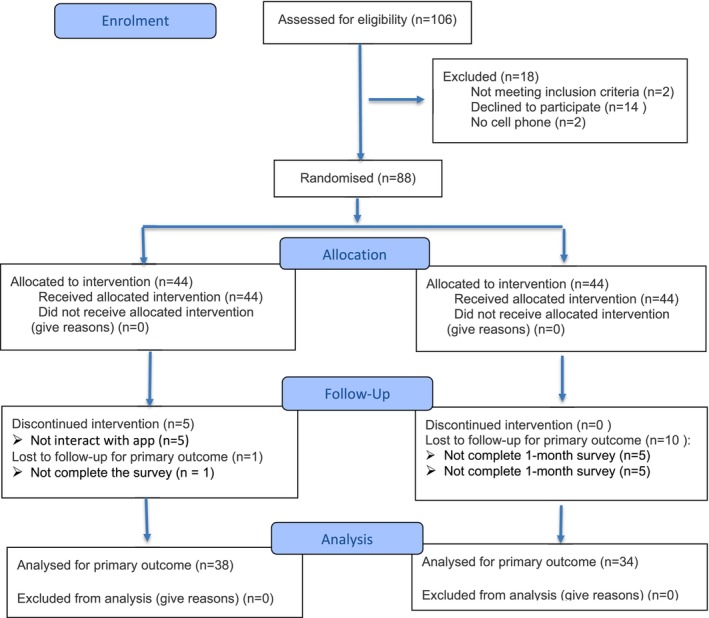
Flow diagram of the progress through the phases of a randomized trial of two groups.

This study was approved by the Chang Gung Medical Foundation Institutional Review Board (No. 202102201B0; approval date: July 16, 2024). Trained research nurses screened participants based on predefined criteria, and all provided written informed consent before participation. Initially, 18 individuals were excluded from the study: 14 declined to participate, two lacked access to a cell phone, and two failed to meet the required health literacy criteria. Upon obtaining informed consent, research nurses collected baseline (T0) data and implemented a randomization procedure to assign 88 participants into two groups: the app group (*n* = 44) and the control group (*n* = 44). During the follow‐up phase, the app group experienced a loss of six participants. In comparison, the control group lost 10 participants, resulting in 38 participants in the app group and 34 in the control group for the final analysis (Figure 2). Data were collected through structured surveys administered at baseline, one month, and three months post‐intervention, ensuring a comprehensive evaluation of participant outcomes across these time points. To mitigate assessor bias, participants completed the questionnaires independently via a secure online survey platform at each interval. This standardized approach minimized potential influences from research staff on participants' responses. All collected data were securely stored in a locked office accessible only to the principal investigator at the data collection site.

### 3.7 Data Analysis

Data were scanned for completeness, and all responses were systematically coded and entered into the IBM SPSS statistics software, version 28. Descriptive statistics were used to summarize the data, calculating frequencies and percentages for categorical variables, and means and standard deviations for continuous variables. The primary outcome analyses included participants who completed all three outcome assessments. Participants who did not complete follow‐up assessments were excluded from the final analysis. Therefore, the analysis was not a full intention‐to‐treat analysis, and no imputation was performed for missing follow‐up data. Generalized estimating equations were used to examine changes in diabetes empowerment, CT‐related critical health literacy, and quality of life across baseline, one month, and three months. The GEE models included group, time, and group‐by‐time interaction terms to determine whether changes over time differed between the app and control groups while accounting for the correlation of repeated measurements within participants. Effect sizes were calculated to estimate the magnitude of the intervention effects for the primary outcomes. Cohen's *d* values were interpreted as small (0.2), medium (0.5), or large effects (0.8) (Cohen 1988). Statistical significance was determined using a two‐tailed alpha level of 0.05.

## 4 Results

### 4.1 Baseline Comparisons Between Groups

Table 1 presents the baseline comparison of demographic and clinical characteristics between the app (intervention) and control groups. Analysis indicated no statistically significant differences in age (*p* = 0.126), duration of diabetes (*p* = 0.211), HbA1c (*p* = 0.127), or fasting blood sugar (*p* = 0.242) between the groups. The gender distribution did not significantly differ (*χ* ^2^ = 1.43, *p* = 0.233), with a slightly higher proportion of males in the control group (58.8%) compared to the app group (44.7%). Although differences in education levels were observed, they were not statistically significant (*χ* ^2^ = 5.364, *p* = 0.147). Similarly, marital status and employment status exhibited no statistically significant differences between the groups (*p* = 0.947 and *p* = 0.106, respectively). While the integration of CT with prescribed medicines approached significance (*χ* ^2^ = 4.74, *p* = 0.094), the majority of participants reported separating the intake of CT and prescribed medications. Furthermore, knowledge of CT ingredients and disclosure of CT use to healthcare professionals showed no statistically significant differences (*p* = 0.279 and *p* = 0.165, respectively). However, a larger proportion of the app group reported knowledge of the ingredients (73.7%) and disclosed their use of CT (84.2%).

**TABLE 1:** Baseline comparisons between the control and app groups (*n* = 72).

Variables	Control group		App group		*T*	*p*
	Mean	SD	Mean	SD		
Age, years	53.4	13.33	58.2	13.09	−1.55	0.126
Duration of diabetes, years	12.9	9.88	10.4	6.48	1.26	0.211
HbA1c	7.34	1.47	6.94	0.60	1.54	0.127
Fasting blood sugar	130.59	36.84	121.92	24.99	1.18	0.242
	***N***	**%**	***N***	**%**	***χ*** ^**2**^	***p***
Gender						
Male	20	58.8	17	44.7	1.43	0.233
Female	14	41.2	21	53.3		
Education						
Primary	6	17.6	3	7.9	5.364	0.147
Secondary	13	38.3	11	28.9		
Bachelor's	10	29.4	21	55.3		
≥ Master's	5	14.7	3	7.9		
Marital status						
Married	18	53.0	22	57.9	0.37	0.947
Single	6	17.6	7	18.4		
Widowed	5	14.7	4	10.5		
Divorced	5	14.7	5	13.2		
Employment						
Employed	17	50.0	22	57.9	2.62	0.106
Unemployed or retired	17	50.0	16	42.1		
Integration of CT and prescriptions						
No change	10	29.4	20	52.6	4.74	0.094
Separated	23	67.6	18	47.4		
Reduced	1	2.9	0	0.0		
Knowledge of CT						
Unknown	13	38.2	10	26.3	1.17	0.279
Known	21	61.8	28	73.7		
Disclosure to healthcare professionals						
No	24	70.6	32	84.2	1.93	0.165
Yes	10	29.4	6	15.8		

### 4.2 Primary Outcomes

**FIGURE 3:**
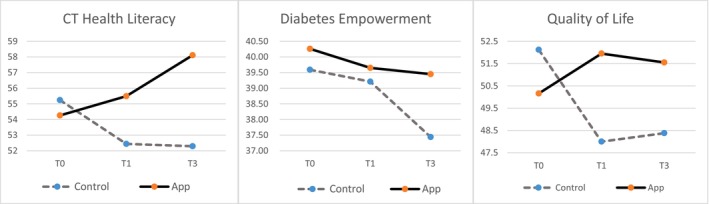
Trends in diabetes empowerment, complementary therapy health literacy, and quality of life across three time points.

Table 2 and Figure 3 present the results from the generalized estimating equations analysis, which evaluated the effects and trends of the intervention on three outcome variables across three time points: diabetes empowerment, CT‐related critical health literacy, and quality of life. For diabetes empowerment, no statistically significant differences were identified between the app and control groups at any of the three time points (all *p* > 0.05), indicating that the intervention did not significantly affect participants' perceived empowerment in diabetes management. However, the trend for the control group was lower empowerment. In contrast, CT‐related critical health literacy demonstrated a statistically significant improvement at T2 compared to baseline (T0) within the app group (Estimate = 6.783, *p* = 0.028), suggesting an increased awareness of risks and benefits associated with CAM over time. Additionally, quality of life significantly improved in the app group at both T1 (Estimate = 6.110, *p* = 0.014) and T2 (Estimate = 5.130, *p* = 0.035) relative to baseline, reflecting the positive impact of the intervention on participants' overall well‐being. Effect‐size analysis showed medium effect sizes, with Cohen's d values at 0.54 for CT‐related critical health literacy and 0.51 for quality of life.

**TABLE 2:** Changes in diabetes empowerment, complementary therapy health literacy, and quality of life across three time points and between groups.

	Control group		App group		Estimate	Std. error	Wald *χ* ^2^	95% Wald CI		*p*
	Mean	SD	Mean	SD				Lower	Upper	
CT health literacy										
Group × T0	55.24	12.44	54.26	14.00	−0.972	3.0728	0.100	−6.995	5.050	0.752
G × T1 vs. T0	52.44	10.08	55.49	12.21	3.941	2.6754	2.170	−1.303	9.185	0.141
G × T2 vs. T0	52.29	11.55	58.11	11.87	6.783	3.0908	4.816	0.725	12.841	0.028
Diabetes empowerment										
Group × T0	39.59	7.81	40.26	6.39	0.675	1.6696	0.163	−2.598	3.947	0.686
G × T1 vs. T0	39.21	6.59	39.65	7.14	−0.019	1.5019	0.000	−2.963	2.924	0.990
G × T2 vs. T0	37.44	7.67	39.45	7.56	1.331	1.6433	0.656	−1.890	4.552	0.418
Quality of life										
Group × T0	52.12	10.10	50.16	10.32	−1.960	2.3746	0.681	−6.614	2.694	0.409
G × T1 vs. T0	48.00	8.36	51.95	10.10	6.110	2.4984	5.980	1.213	11.006	0.014
G × T2 vs. T0	48.38	9.05	51.55	11.13	5.130	2.4360	4.435	0.356	9.904	0.035

### 4.3 Harms or Unintended Effects

No safety concerns or adverse effects were reported during the trial.

## 5 Discussion

This study advances the field by developing and evaluating the Educational App for Complementary Therapy in Diabetes Management (ECT‐Diabetes App), an innovative, user‐centred digital intervention designed to improve CT‐related critical health literacy, particularly participants' ability to evaluate CT information, consider potential safety risks, and make more informed decisions about CT use alongside conventional diabetes care, without promoting specific CT modalities. In response to the three hypotheses, the intervention group (1) exhibited greater improvement in CT‐related critical health literacy; (2) demonstrated no statistically significant difference in diabetes empowerment scores; and (3) reported improved perceived quality of life compared with the control group. Although empowerment outcomes did not reach statistical significance, the observed improvements in CT‐related critical health literacy and quality of life underscore the app's educational value and psychosocial relevance. The results suggest that digital tools incorporating empowerment strategies, operationalized through CT‐related information appraisal, safety evaluation, and benefit–risk assessment skills, can enhance individuals' understanding of CT and provide more confident, evidence‐based decision‐making in chronic disease management.

Consistent with Hypothesis 1, the statistically significant improvement in CT‐related critical health literacy underscores the app's effectiveness in addressing a previously underexplored area of diabetes education: the critical appraisal of CT‐related information and assessment of potential safety concerns. A key feature contributing to this result is the app's integrated benefit–risk assessment module (Chang et al. 2021), which guides individuals in evaluating CT interventions in relation to their own medical profiles. By engaging with scenario‐based quizzes and interactive decision‐making activities, users can apply newly acquired knowledge in a personalized and reflective manner (Chang and Huang 2025). Its core mechanisms were information appraisal, credibility evaluation, risk recognition, and structured decision‐making. This aligns with Tian et al.'s (2025) conceptualization of critical health literacy as involving the ability to critically evaluate health information and apply it within social and cultural contexts. Although directly comparable studies are lacking, these results align with systematic reviews highlighting the positive impact of digital health interventions on patient education and engagement (Naef et al. 2023; Verweel et al. 2023).

Hypothesis 2 was not supported, as no statistically significant improvement was observed in diabetes empowerment. This contrasts with meta‐analytic evidence showing that empowerment interventions for diabetes can produce small but significant effects, particularly when targeting diabetes control and self‐efficacy (Chen et al. 2021). Several factors may explain this result. First, empowerment is a multidimensional construct that often requires sustained engagement to produce measurable changes in self‐efficacy, decision‐making autonomy, and behavioural adaptation (Duarte‐Diaz et al. 2023). Compared with the more intensive and longer interventions included in prior meta‐analyses, the present intervention, with one‐month and three‐month follow‐up, may have been insufficient to generate broader empowerment effects. Second, the diabetes empowerment measure may not have fully captured the app's CT‐specific focus. The ECT‐Diabetes App targeted CT‐related critical health literacy and decision‐making through features such as credibility evaluation, product safety assessment, and benefit–risk algorithms, whereas the diabetes empowerment scale assessed broader self‐efficacy and perceived control in diabetes management. As Brew‐Sam and Chib (2019) noted, theoretically empowering app features may have limited behavioural impact without sufficient customization, social support, or patient–provider communication. In addition, CT‐related decision‐making is also shaped by expected benefits, perceived safety, dissatisfaction with conventional medicine, social influence, accessibility, and cultural or traditional beliefs (Tangkiatkumjai et al. 2020), which may affect whether users apply app‐based knowledge in practice. Future versions should include usage tracking, tailored feedback, guided reflection, and stronger communication or support functions to better translate app engagement into empowerment outcomes.

Consistent with Hypothesis 3, the observed improvement in quality of life may be attributed to participants' increased confidence in evaluating CT options based on their individual needs and making safer, evidence‐based decisions, thereby increasing satisfaction with diabetes self‐management. This result is supported by existing literature that reports the positive effects of digital health interventions on health outcomes, particularly those that incorporate interactive and personalized features (Hummel et al. 2022; Ossenbrink et al. 2023). Nonetheless, other studies have reported mixed results, with some interventions having limited or no impact on quality of life or self‐management behaviours (Doupis et al. 2020). These inconsistencies underscore the critical role of thoughtful intervention design, user engagement, and integration in an empowerment process framework. The ECT‐Diabetes App's focus on individualized learning and active participation through benefit–risk assessments may distinguish it from less interactive or more generic tools. Notably, the quality of life was self‐reported and not a direct health outcome; therefore, our findings should be interpreted within this limitation.

### 5.1 Limitations, Strengths and Recommendations for Further Research

Several limitations should be acknowledged. First, potential selection bias may have arisen from the restrictive inclusion criteria, which required participants to own a smartphone, read Mandarin, and meet a minimum level of health literacy. These prerequisites likely excluded older adults, individuals from lower socioeconomic backgrounds, and participants with limited digital or health literacy—groups who are typically underserved yet stand to benefit considerably from digital health interventions. Consequently, the sample may not sufficiently represent the broader diabetic population, limiting generalizability. Future research should adopt more inclusive recruitment strategies, such as multilingual support, alternative formats for users with low literacy, and accommodations for individuals with age‐related challenges in IT usability.

Second, attrition bias is a concern, given the 18.2% participant dropout rate. Despite efforts to ease participation through online self‐administered surveys and monthly reminders, those who completed the study may have been more motivated or better equipped, potentially due to higher health literacy or increased engagement, which could have inflated estimates of intervention effectiveness. Additionally, the absence of usage analytics (such as login frequency and duration) limited insight into participant engagement and adherence. Addressing these challenges in future studies may involve real‐time tracking, automated prompts, personalized feedback, and incentives for survey completion. Implementing longer interventions, reinforcement mechanisms (such as peer support or guided reflection), and longitudinal assessment of empowerment trajectories is also recommended.

Despite these limitations, this study offers valuable insights into the efficacy of digital interventions for improving CT‐related literacy and quality of life in diabetes management. Through principles of sound instructional design, a user‐centred development, and a robust process framework, the ECT‐Diabetes App fills a critical gap in chronic disease self‐management. Although a longer follow‐up may be necessary to fully capture changes in empowerment, the app has substantial potential to support safer CT‐related decision‐making by helping participants appraise CT information, evaluate the benefit–risk of CT use, and promote communication with healthcare professionals.

### 5.2 Implications for Policy and Practice

The implementation of the Educational App for Complementary Therapy in Diabetes Management holds promise in supporting clinical practice in diabetes management. By providing individuals living with diabetes with accessible, evidence‐based information on CT, the app enhances patient health education to support informed appraisal, benefit–risk evaluation, and communication with healthcare professionals about CT use alongside conventional diabetes care. The result is likely to cultivate more active personal engagement in care planning, potentially leading to improved adherence to prescribed therapies. Moreover, the app fosters improved communication between patients and healthcare providers by equipping them with the requisite knowledge necessary to engage in substantive discussions about CT use. This interaction can lead to the development of more coordinated and personalized care plans, thereby reducing the risk of adverse interactions between conventional treatments and CT. The emphasis on improving CT‐related critical health literacy addresses a critical deficiency in diabetes education, enabling individuals to critically evaluate CT options and discern credible information sources. Although this study was conducted within a specific cultural context, the underlying principles of health literacy and quality of life possess more generalized applicability. Adapting the app's content to accommodate various languages and cultural contexts could extend its benefits to a wider population of individuals managing diabetes, thereby enhancing the overall quality of diabetes care across diverse settings.

## 6 Conclusion

Despite high prevalence rates of CT use among individuals diagnosed with diabetes, there is limited guidance on how to effectively assess its benefits and risks or communicate its usage with healthcare providers. This study demonstrates that digital education tools can effectively enhance knowledge about the benefits and risks of CT use in diabetes care, supporting its use in promoting quality of life. The Educational App for Complementary Therapy in Diabetes Management demonstrates the feasibility and effectiveness of integrating CT‐related critical health literacy into routine diabetes care, establishing a robust foundation for future large‐scale or longitudinal research. By aligning user needs with theoretical rigour, this intervention advances academic discourse on CT‐related critical health literacy and provides a practical digital strategy for helping individuals with diabetes critically evaluate CT information, communicate with healthcare professionals, and make safer, evidence‐informed decisions within an integrative care framework.

## Author Contributions

Study conception and design: H.‐Y.C. Data collection: K.‐C.Y., Y.‐Y.H., J.‐H.L., F.‐H.L. Data analysis and interpretation: H.‐Y.C. Drafting of the article: H.‐Y.C. Critical revision of the article: H.‐Y.C., Y.‐Y.H., K.‐C.Y., J.‐H.L., F.‐H.L. All authors read and approved the final manuscript.

## Funding

This study was supported by the National Science and Technology Council, Taiwan (NSTC 111‐2314‐B‐255‐002‐MY3 and 114‐2314‐B‐255‐014‐MY3).

## Ethics Statement

The study protocol was approved by Chang Gung Medical Foundation Institutional Review Board (No. 202102201B0).

## Conflicts of Interest

The authors declare no conflicts of interest.

## Data Availability

The data that support the findings of this study are available from the corresponding author, H.Y.C., upon reasonable request.
